# Scalability of Sartobind^®^ Rapid A Membrane for High Productivity Monoclonal Antibody Capture

**DOI:** 10.3390/membranes13100815

**Published:** 2023-09-27

**Authors:** Sabrina Yang, Ryszard Braczkowski, Shih-Hsun Chen, Ricarda Busse, Yudhi Li, Louis Fabri, Innocent Berbelle Bekard

**Affiliations:** 1 CSL Innovation Pty Ltd., 655 Elizabeth Street, Melbourne, VIC 3000, Australia; 2 Sartorius Stedim Biotech GmbH, August-Spindler-Strasse 11, 37079 Goettingen, Germany; 3 Sartorius Stedim Singapore Pte Ltd., 30 Pasir Panjang Rd., #06-31A/32, Singapore 117440, Singapore

**Keywords:** monoclonal antibody, protein A, membrane chromatography, bioprocessing, rapid cycling, process intensification

## Abstract

Improved upstream titres in therapeutic monoclonal antibody (mAb) production have shifted capacity constraints to the downstream process. The consideration of membrane-based chromatographic devices as a debottlenecking option is gaining increasing attention with the recent introduction of high-capacity bind and elute membranes. We have evaluated the performance and scalability of the Sartobind^®^ Rapid A affinity membrane (1 mL) for high-productivity mAb capture. For scalability assessment, a 75 mL prototype device was used to process 100 L of clarified cell culture harvest (CH) on a novel multi-use rapid cycling chromatography system (MU-RCC). MabSelect™ PrismA (4.7 mL) was used as a benchmark comparator for Protein A (ProtA) resin studies. Results show that in addition to a productivity gain of >10×, process and product quality attributes were either improved or comparable to the benchmark. Concentrations of eluate pools were 7.5× less than that of the benchmark, with the comparatively higher bulk volume likely to cause handling challenges at process scale. The MU-RCC system is capable of membrane operation at pilot scale with comparable product quality profile to the 1 mL device. The Sartobind^®^ Rapid A membrane is a scalable alternative to conventional ProtA resin chromatography for the isolation and purification of mAbs from harvested cell culture media.

## 1. Introduction

Since the advent of therapeutic monoclonal antibodies (mAbs) in 1975, packed-bed chromatography remains the predominant technology of choice for the isolation and purification of these life-saving proteins [1,2]. The robustness of this technology is utilised as a standardised purification scheme to many mAbs derived from cell culture production systems. The pressure to accelerate drug development has challenged process scientists and vendors to continuously improve approaches to shorten product development timelines, ideally at reduced cost with little or no impact on product quality. This is observed in the upstream process, where improvement efforts resulting in approximately five-fold increases in mAb titres have been realised [3,4,5]. This increase in product yield has shifted the bottleneck of the production process from upstream to downstream, principally the mAb capture step [4,6].

Protein A (ProtA) resins are the mainstay for mAb capture and can constitute more than 50% of the downstream cost of mAb production [7,8,9]. The focus on this step for efficiency gains in the downstream process can have a significant impact. Attributes including dynamic binding capacity, resin lifetime, and resin capacity utilisation are key determinants of the cost of mAb production and the measure of the efficiency and value of the process step. As with many resin-based chromatography media, the narrow pore size range 60–120 nm of the ProtA resin can be impacted by diffusional mass transfer for mAb capture [8]. Hence, operation at residence times in the order of minutes is required to attain the desired pressure drop and load density, resulting in extended cycle times.

The emergence of membrane-based and fibre-based capture chromatography, where mass transfer is governed primarily by convection, can be a rapid process due to the micron-size pores and consequent binding site accessibility of these devices [10,11,12]. However, mAb binding capacity is negatively impacted by convection mass transfer. This limitation appears to have been overcome via recent improvements in ProtA chromatographic membrane and fibre devices [13,14,15]. Some improvements combine both convection and diffusion mass transfer mechanisms to attain mAb binding capacities comparable to those of conventional resins (>30 g/L), but with a significant reduction in residence time (mins to s) [15]. This is made possible by the relatively wider pore size range, 3–5 µm, of the base matrix, which supports convection, and porous supports featuring 2–3 µm diffusive regions, which improves mAb binding capacity. With cycle times of <20 min compared to hours, productivity gains more than 10× that of packed bed chromatography have been reported [14,15,16]. The key value proposition of this technology is the full capacity utilisation of the membrane in a single instance of batch purification, which can have a positive impact on the cost of goods sold.

There are additional benefits in using an affinity membrane for mAb capture, such as a reduction in facility footprint and the elimination of column handling and its associated bioburden and batch-to-batch cross contamination risks. The short cycle times would mean that the duration of process development and characterisation campaigns could be significantly reduced. For example, a 100-cycle membrane lifetime study could be completed in <40 h on a chromatographic membrane, compared to four weeks on a packed bed column.

In this study, we assessed the performance and scalability of the Sartobind^®^ Rapid A affinity membrane for mAb capture. To our knowledge, this was the first time the new Rapid A membrane has been used in combination with the novel MU-RCC at pilot scale to process 100 L of CH. MabSelect™ PrismA was used as a benchmark for ProtA resins.

## 2. Materials and Methods

### 2.1. ProtA Chromatography Devices

The ProtA chromatography membrane device used in this study was the Sartobind^®^ Rapid A convective-diffusion membrane (Sartorius, Göttingen, Germany). A 1 mL membrane volume (MV) device (3 mL hold-up volume) was used for initial development work, with scaleup assessment conducted on a 75 mL device (200 mL hold-up volume). A fixed bed height of 4 mm applied to both membranes.

ProtA resin MabSelect™ PrismA resin (Cytiva, Uppsala, Sweden) was purchased in a HiScreen™ chromatography column format (10 cm bed height and 4.7 mL bed volume). This format is a good model for process-scale columns based on our experience. The resin was used as a benchmark reference for membrane chromatography.

### 2.2. Monoclonal Antibody

A model human IgG4 mAb with pI 8.6 was used in this study. The mAb was expressed in a proprietary Chinese hamster ovary (CHO) cell line (Melbourne, Australia) in suspension culture using a stirred-tank fed-batch approach. CH was frozen at −80 °C and thawed as required for the capture chromatography experiments. Four batches of comparable CH derived from 5 L to 500 L bioreactors were used in this study (Table 1). Bench scale experiments were performed with CH batches CH1, CH2, and CH3, whereas CH4 and CH5 were used for the pilot scale runs.

### 2.3. Buffers and Reagents

The buffers used in the purification scheme are proprietary to CSL Innovation Pty Ltd. (Melbourne, Australia). However, results similar to what is presented in this paper can be achieved using the buffers published elsewhere for the purification of different mAb isotypes using the Sartobind^®^ Rapid A membrane device [15,17]. The designation, application, and pH ranges of the solutions used in this study are listed in Table 2. Chemicals for the preparation of chromatography solutions were sourced from Merck (Darmstadt, Hesse, Germany).

### 2.4. Analytical Tools

#### 2.4.1. Protein Concentration Using ProtA-HPLC

Protein concentrations of CH and flowthrough samples were determined through analytical high-performance affinity chromatography (ProtA-HPLC) using a POROS™ ProteinA 20 µm column (2.1 mm ID × 30 mm) with an UltiMate 3000 System from ThermoFisher Scientific (Waltham, MA, USA) equipped with a UV detection system. The reference standard used was a bulk purified GMP-grade version of the model mAb used in this study. The tests were performed at 2.0 mL/min, with buffer A—35 mM Tris, 150 mM NaCl, pH 7.8—and buffer B—50 mM Citrate, 250 mM NaCl, pH 3.0—and a sample application of 50 µg. Samples of the purified protein (eluate pool) were tested with ProtA-HPLC. Results from this test were used for mAb yield calculations.

#### 2.4.2. Monomer and Aggregate Determination via Size Exclusion HPLC

Monomer and aggregate levels were determined through analytical size exclusion chromatography (SEC-HPLC) using an ACQUITY UPLC BEH200 SEC 1.7 µm column (4.6 mm ID × 150 mm) from Waters with an UltiMate 3000 System from ThermoFisher Scientific. The runs were performed at 0.2 mL/min, with 20 µg sample application and 100 mM MOPS, 210 mM NaCl, pH 7.0 mobile phase. The elution profile was monitored at 280 nm. The proportion of monomer and aggregate in the analytes was estimated through integrating the peak areas of the early-eluting aggregate peak(s), late-eluting fragment peak(s), and the monomer peak.

#### 2.4.3. Host Cell Protein and Residual ProtA via Enzyme-Linked Immunosorbent Assay (ELISA)

The host cell protein (HCP) concentration of product pools was measured using a CHO HCP 3rd Generation ELISA Kit F550-1 (Cygnus Technologies, Leland, NC, USA). The HCP concentration of the analytes was normalized to mAb concentration, from which the HCP log reduction value (LRV) across the capture step was calculated. A ProtA ELISA Kit (9333-1) from Repligen, designed for the detection and quantitation of residual MabSelect SuRe^TM^ ligand, was used for the detection of leached ProtA from the chromatographic devices.

#### 2.4.4. HCP Identification using Mass Spectrometry (MS)

HCP species were identified in product pools using liquid chromatography–tandem mass spectrometry (LC-MS/MS). Data collection was performed using a ThermoFisher Scientific UltiMate 3000 HPLC connected to a Bruker Qq-TOF Impact II MS. Sample preparation was performed for 100 µg of total protein, which underwent trypsin digestion (ThermoFisher Scientific, SMART soluble), followed by dithiothreitol (DTT) reduction, iodoacetamide alkylation, and DTT neutralization. Buffer A was 0.1% formic acid (FA) in water and Buffer B was 0.1% FA in acetonitrile. For analysis, 32 µg of sample was injected and separated using a Waters CSH™ 2.1 mm × 100 mm, C18, 2.5 µm, 130 Å ultra-high-performance liquid chromatography column at a flow rate of 0.2 mL/min and with a gradient from 3 to 30% Buffer B over 46 min with a 40 °C column temperature. The MS was operated in electrospray ionisation (ESI) positive ion mode, with an MS1 and MS2 scan range of 100–2200 *m*/*z*. The eluting peptides were ionised using ESI and fragmented via collision-induced dissociation. The resulting MS spectra were analysed using ProteomeDiscoverer 2.1, where the Sequest HT search engine was used to interrogate the data against a concatenated target and decoy database. The database included the reference sequence of the CHO proteome (RefSeq Assembly Accession: GCF_000223135.1), a list of common contaminating proteases and sample handling proteins (cRAP database), sequences of the IgG4 mAb, and reversed sequences for all entries. Peptide spectral matches (PSMs) were grouped by protein and considered a true positive if the PSMs and the protein had a less than or equal to 1% chance of being a false positive (false discovery rate ≤ 1%), and if the protein was identified with ≥2 unique peptides in duplicate preparation of the same sample.

#### 2.4.5. hcDNA via qPCR

Host cell DNA (hcDNA) was quantified using the highly sensitive resDNASEQ™ Quantitative CHO DNA Kit (PCR) from ThermoFisher Scientific (Waltham, MA, USA).

### 2.5. Chromatography Equipment

A Cytiva ÄKTA Pure 25 chromatography system was used for bench-scale experiments with the Sartobind^®^ Rapid A membrane (MV = 1.0 mL) and MabSelect™ PrismA column (Column Volume, CV = 4.7 mL). UV monitor (U9-M) was measured using simultaneous triple wavelength detection at 10 Hz with a 2 mm flow cell pathlength (280 nm). Sartorius’ prototype MU-RCC skid was used for scale-up experiments with Sartobind^®^ Rapid A (MV = 75 mL). The skid utilises two UV detectors positioned upstream and downstream of the chromatography device. Both UV flow cells are 2 mm in pathlength with a detection frequency of 4 Hz. A flow rate of 750 mL/min was used for the Rapid A pilot device.

### 2.6. Dynamic Binding Capacity Measurement

Dynamic binding capacity (DBC) of the chromatographic devices at 10% mAb breakthrough (DBC_10%_) was assessed on the ÄKTA Pure 25 chromatography system using CH1 as feedstock. For the Rapid A membrane, product load residence times of 0.14, 0.20, 0.33, and 1.0 min were studied. For the MabSelect™ PrismA resin, residence times of 2, 3, 4, and 6 min were studied. To ensure product breakthrough, the membrane was loaded to 90 g/L, whereas the MabSelect™ PrismA resin was loaded to 73 g/L. The mAb breakthrough was assessed using two complementary approaches.

First, flowthrough fractions were collected during the loading phase and analysed for mAb concentration for the estimation of 10% mAb breakthrough.

The second approach was based on step changes in the UV-280 nm chromatogram. Here, the 100% breakthrough absorbance (UV_max_) was determined through bypassing the column and recording the intensity of the UV-280 nm signal at steady state. The steady state UV reading during product load (UV_offset_) was used as baseline for contaminant offset. From the UV profile of the DBC_10%_ experiment, the volume (V_x_) during loading, at which the UV reading was approximately 10% of the difference between UV_max_ and UV_offset_, was estimated and used in the DBC_10%_ calculation as follows:

(1) $${DBC}_{10\%}=\frac{(V_{x}-V_{0})\times \left[C\right]}{V_{membrane|column}}$$

where V_0_ is the holdup volume of the system and device, C is the mAb concentration of the CH, and V_membrane|column_ is the volume of membrane or resin in the chromatographic device.

### 2.7. Determination of Productivity

Productivity is described as the rate of mAb production per litre of chromatography media. The productivity of the chromatographic devices was calculated using the following relationship:

(2) $$Productivity=\frac{Mass\;of\;purified\;mAb\;per\;cycle}{V_{membrane|column}\times Cycle\;time}$$

The unit of productivity is gram of mAb per L of chromatography media per hour (g/L/h).

### 2.8. ProtA Capture Chromatography

The chromatography sequence for mAb capture from the CH, using the various chromatographic systems and devices, is summarized in Table 3.

## 3. Results

### 3.1. Increased Productivity—Comparable DBC, Yield, and Fast Cycling Time

**Figure 1:**
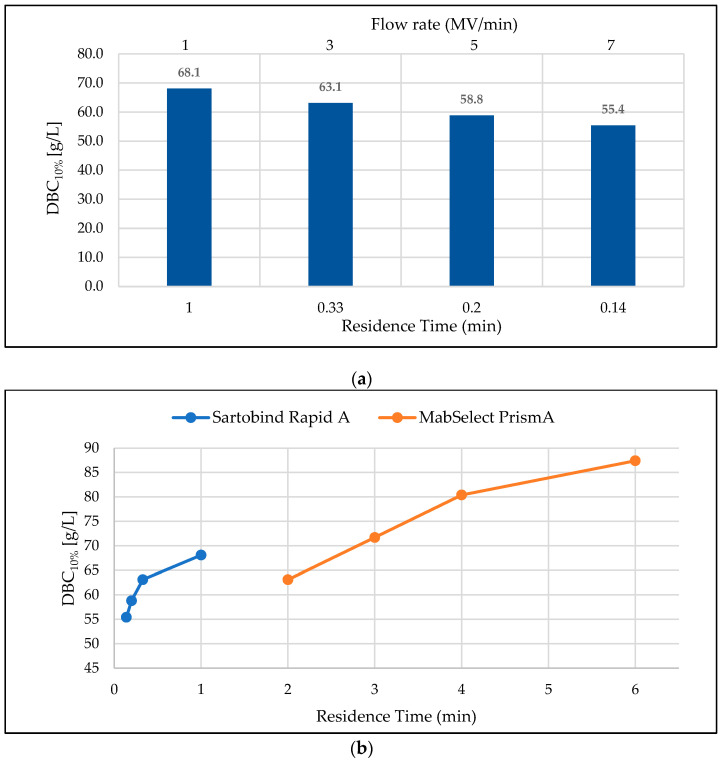
(**a**) DBC_10%_ of Sartobind^®^ Rapid A membrane at selected flow rates and corresponding residence times. (**b**) Comparison of DBC_10%_ of Sartobind^®^ Rapid A membrane (1 mL) and MabSelect™ PrismA resin at selected residence times. CH1 was used. The DBC was calculated based on UV breakthrough curves.

**Figure A1:**
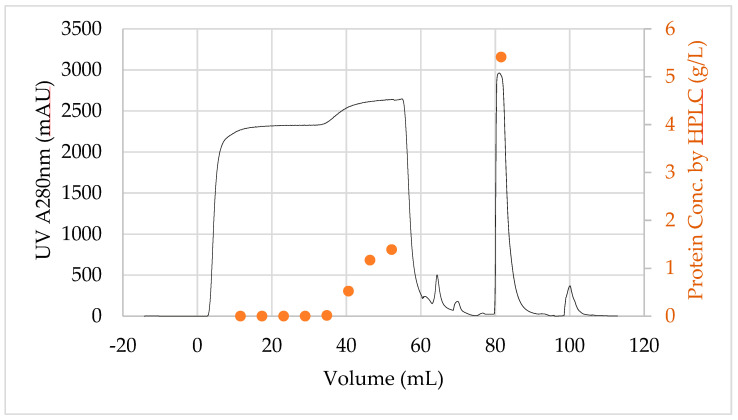
mAb breakthrough detected via ProtA-HPLC in flowthrough fractions from the Sartobind^®^ Rapid A membrane device at a product load flow rate of 5 MV/min. The feedstock used was CH. The UV A280 nm chromatogram is shown for reference. The flowthrough fractions were collected at 10 g/L intervals (~5.8 mL per fraction).

Figure 1 compares DBC_10%_ for the membrane and resin chromatographic devices (based on UV breakthrough curves) at vendor-recommended operating flow rates considering process-scale application. For residence times of ≤1 min, the DBC_10%_ for Rapid A showed a dependence on flow rate and ranged from 55.4 g/L to 68.1 g/L (Figure 1a) [9]. The MabSelect™ PrismA resin showed a similar dependence of DBC_10%_ on residence time (2–6 min), ranging from 63.1 g/L to 87.4 g/L (Figure 1b) [8]. The UV-based DBC_10%_ results were complemented by mAb detection in flowthrough fractions via ProtA-HPLC assay. Representative results for this approach, using the 5 MV/min flow rate, are shown in Figure A1, in Appendix A.

Based on process-scale considerations, including the pressure limits, productivity, and capacity utilisation of chromatography media, the operational product load residence time selected for Rapid A was 0.2 min, and that for MabSelect™ PrismA was 4 min. Table 4 shows the corresponding load densities on these devices, including product and process attributes following a typical purification cycle using the sequence described in Table 3. The yield on the membrane was lower compared to the resin, which may be a function of the elevated elution pH of 3.6 and reduced contact time on the membrane. The partitioning of impurities including aggregates, HCP, and hcDNA, as well as leached ProtA ligand, was significantly better on the membrane. This was reflected in the >99% monomer purity and the single HCP species identified using mass spectrometry, which was detected to be below the 50 ppm threshold. With the reduced cycle time, the productivity on the membrane was circa 11× higher than that of the resin. The concentration of product pool from the membrane device was approximately 7.0× less than that of the MabSelect™ PrismA resin, which is reflected in the 11 MV equivalent of eluate pool and could be a function of the mAb desorption kinetics on the membrane.

### 3.2. Robustness of Rapid A Membrane over 100 Repeated Cycles

**Figure 2:**
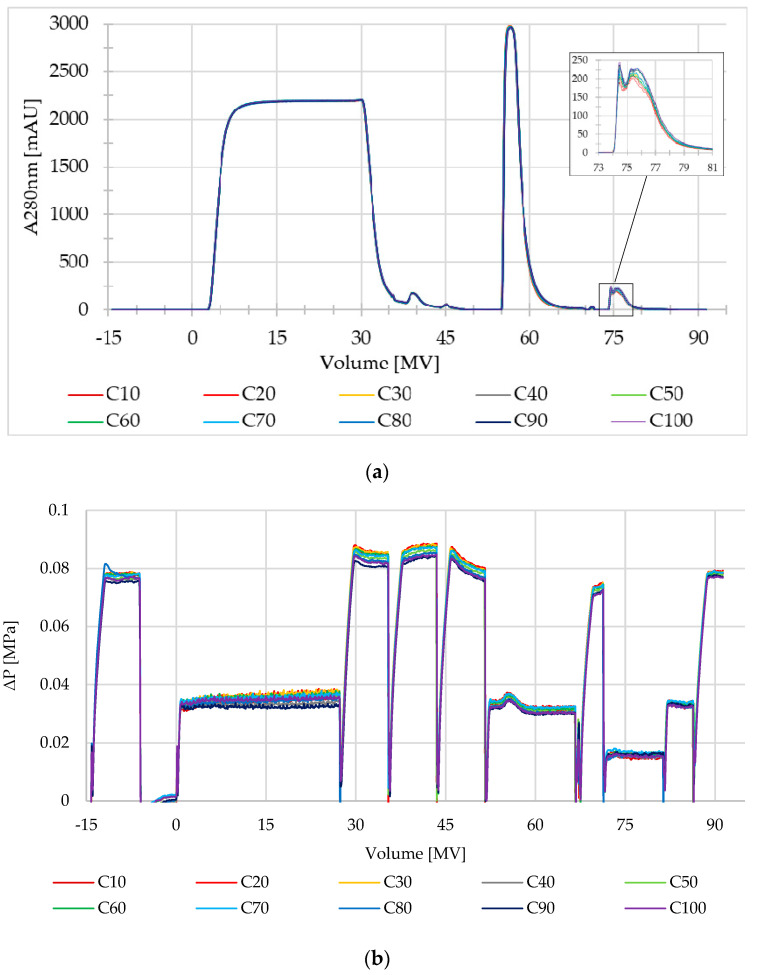
(**a**) Overlay of UV280 nm profiles for 100 cycles of capture chromatography by Sartobind^®^ Rapid A membrane (1 mL) at a load density of 45 g/L using feedstock batch CH3 and CH4. Profile for every 10th cycle is shown. The insert shows a close-up of the regeneration peak. (**b**) Overlay of corresponding delta pressure (ΔP) profiles of every 10th cycle of capture chromatography on the Sartobind^®^ Rapid A membrane (1 mL).

Recycling of the Rapid A membrane was assessed via performing 100 purification cycles on a 1 mL device and monitoring selected performance indicators including UV-280 nm traces, pressure drops, and quality of the purified material. Figure 2a shows UV-280 nm profiles from the lifetime runs in 10-cycle increments, which overlapped neatly. The UV absorbance reading remained at baseline during the equilibration of the membrane (−15 to 0 MV), stayed elevated during the product loading phase up to about 25 MV, and dropped close to baseline during the three post-load wash phases, targeting unbound proteins and impurities, up to about the 55 MV mark. The product elution phase at low pH was marked by a relatively sharp UV peak at about 60 MV, with the membrane regeneration peak appearing at 75 MV. The marginal changes observed for the regeneration peak show an effective cleaning regimen, which is critical for membrane lifetime. The pressure drop across the membrane over 100 cycles was unremarkable (Figure 2b), ranging from 0.03 to 0.04 MPa during the product loading phase and 0.07 to 0.09 MPa during the equilibration and wash phases where a high flow rate (10 MV/min) was applied.

**Figure 3:**
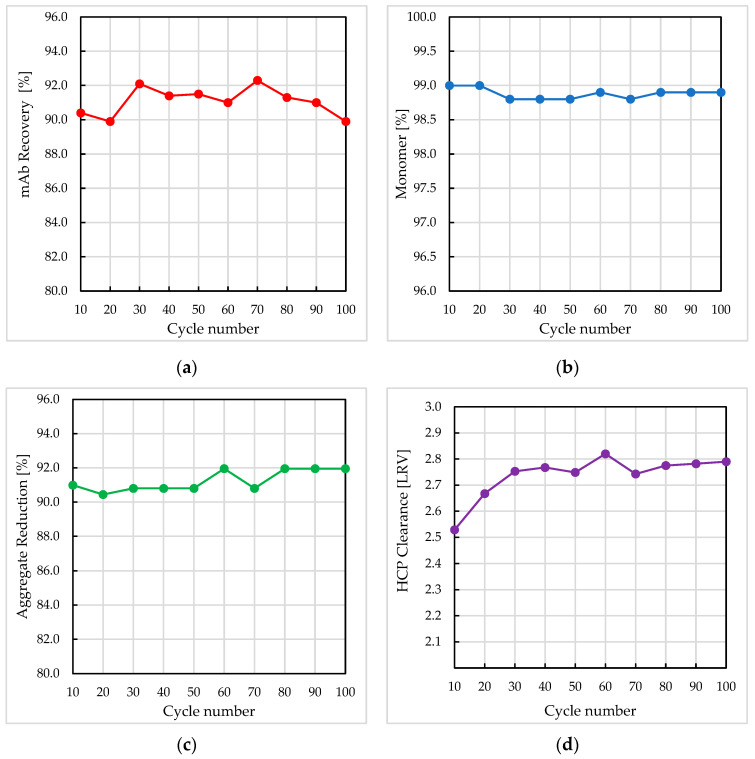
Performance of Sartobind^®^ Rapid A membrane over 100 purification cycles. Data for every 10th cycle is shown for (**a**) mAb recovery (%), (**b**) onomer (%), (**c**) aggregate reduction (%), and (**d**) HCP clearance log reduction value (LRV).

**Figure A2:**
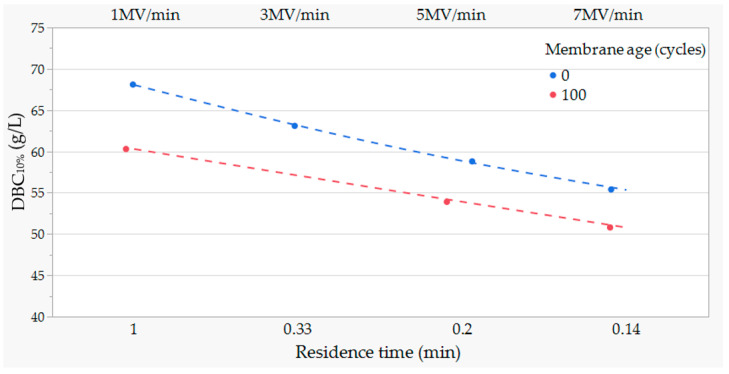
Assessment of DBC_10%_ before use and post 100 cycles. The DBC was calculated using UV breakthrough curves.

Figure 3 shows the performance of the Sartobind^®^ Rapid A membrane (1 mL) against selected process and product attributes over the 100-cycle membrane lifetime runs. The robustness of the membrane was demonstrated through showing marginal change in key performance indicators including product recovery, monomer purity, aggregate reduction, and HCP clearance over 100 cycles. Here, monomer purity remained above 98% with a consistent product recovery ranging between 90% and 92%. Coupled with an HCP LRV of >2.5, the eluate pools from the Rapid A membrane appear to have less impurity burden compared to the benchmark (Figure 3, Table 4). A check of the DBC_10%_ of the membrane device, before and after the lifetime study, showed circa 8.3% decrease in binding capacity at the optimal product load residence time of 0.2 min (Figure A2, in Appendix A).

### 3.3. Scalability of Sartobind^®^ Rapid A Membrane—Purification of 100 L CH with MU-RCC Chromatography System

A 75 mL prototype Rapid A membrane was used to assess scalability of the performance observed at the 1 mL scale. Two lots of 50 L CH (CH4 and CH5), with a mAb titre of circa 1.8 g/L, were purified on this membrane using the MU-RCC chromatography system as per the method sequence in Table 3. To our knowledge, this was the first time the MU-RCC system has been used to process a relatively large volume of mAb material at pilot scale.

**Figure A3:**
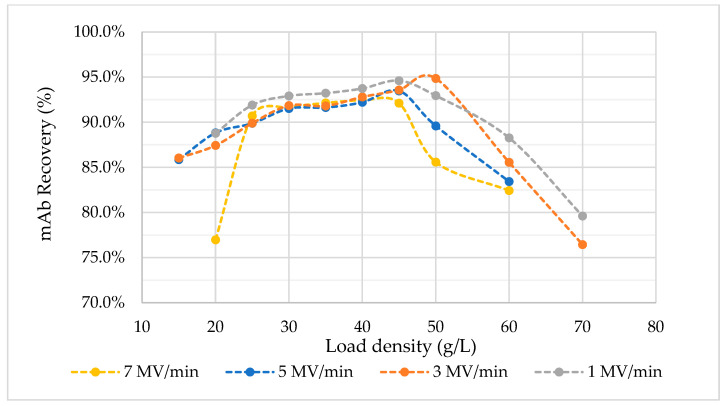
Impact of load density and flow rate on product recovery. The study was performed using the 1 mL membrane device.

Approximately 10% of product introduced onto the membrane was detected in the post-load-wash fraction when the first 50 L of CH4 was processed at a load density of 45 g/L. Here, 26 purification cycles were run on the membrane, returning an average yield of circa 81%, which differed from the ~90% yield observed on the 1 mL device. The load density was likely at the limits of the membrane device (Figure A3, in Appendix A) and so the purification sequence was adjusted to target 35 g/L for the second lot of CH5; as a result, 90% recovery was achieved.

**Figure 4:**
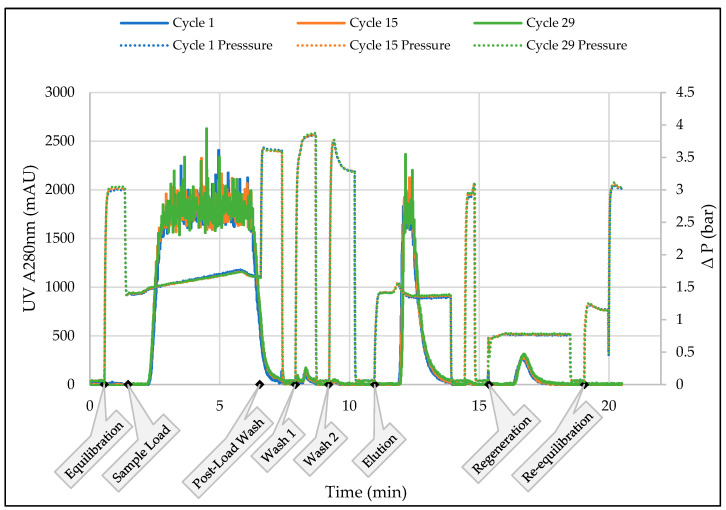
Overlay of UV-280 nm profile of the start, middle, and end cycles of capture process for 50 L of CH5 with Sartobind^®^ Rapid A 75 mL using MU-RCC unit. The call-outs on the x-axis show the start of the various phases of the chromatography sequence.

Figure 4 shows an overlay of UV-280 nm profiles for 3 out of the 29 purification cycles for the second 50 L purification episode at 35 g/L load density. The profiles showed good reproducibility. The UV profile was similar to that observed at the 1 mL scale save for the noise in the signal during the product loading and elution phases, which was a function of UV detector saturation. Product quality and recovery were comparable between the 1 mL and 75 mL membrane devices (Table 5).

## 4. Discussion

The application of chromatographic membrane devices in therapeutic protein purification is not novel [10,18]. The binding capacity limitations of these devices in comparison to resins have restricted commercial usage to the more economically viable flowthrough operation. Recent improvements in the DBC of membrane formats offer an attractive integrative solution for processing high-titre cell culture preparations within an acceptable process duration. This was the main driver for assessing the performance and scalability of the Sartobind^®^ Rapid A affinity membrane. In addition to the benefits of chromatographic resins, membranes may offer significant productivity gains, flexible linear scale-up, and small facility footprint. The selection of MabSelect™ PrismA resin as a benchmark for ProtA affinity adsorbents is based on our experience with regular assessments of vendor offerings and scale-up of ProtA adsorbents for mAb capture.

The comparatively higher DBC_10%_ observed for the PrismA resin is a complement of extended residence time to its diffusion-driven mass transfer properties [8] and is expected to drop precipitously with increasing flow rate [19]. It is interesting, though, that the 63.1 g/L DBC_10%_ achieved at the shortest residence time (2 min) operated on the PrismA resin was attainable on the membrane device at 6× shorter residence time (0.33 min). Indeed, for the protein load residence times studied, the membrane was operated at flow rates up to 9× faster than that of the resin.

The performance of Sartobind^®^ Rapid A membrane in mAb capture has previously been reported [9,15]. The characterisation of HCP species in effluents from the smaller membrane device via mass spectrometry, and the use of an IgG4 isotype in this work, provides additional insights to previous work [15,17]. Briefly, the DBC and impurity profile of the eluate pools from the Rapid A device show comparable results even for the different mAb isotypes and different process solutions applied in these studies. Other affinity-based fibre and membrane devices with improved mAb binding capacities have also been reported [14]. The work reported here is, to our knowledge, the first assessment of the Rapid A device at pilot scale using the MU-RCC membrane chromatography system and its comparison to smaller-scale devices.

The exploitation of both convection and diffusion mass transfer mechanisms in the design of the Rapid A membrane appear to have overcome the DBC limitation, which to date has previously challenged affinity-based chromatographic membranes in protein purification [15]. With DBC_10%_ in excess of 55 g/L at residence times as short as 8.4 s, the challenges of processing high-titre cell culture fluid may be overcome. For an affinity membrane, these values are a significant improvement compared to those previously reported [9]. The data reported here demonstrate that the membrane-based adsorbent may be more effective in the partitioning of process- and product-related impurities including hcDNA, HCP, and aggregates from the target mAb, consistently delivering mAb purities of >98% over a lifetime of 100 cycles. This could potentially reduce the number of subsequent polishing steps required in a mAb purification scheme, depending on the expected quality target product profile. The apparent improvement in HCP clearance on the membrane with cycle number is likely due to a reduction in non-specific binding of HCP to the base matrix. We have observed a similar trend for ProtA resins. It has been reported that the Rapid A membrane could be re-used for up to 200 cycles for the one batch, as trends of key performance indicators including mAb recovery, purity, and pressure drop across the membrane only showed marginal shifts from initial performance for the different mAb isotypes studied [15]. The 8.3% drop in the DBC of the membrane following 100 repeated uses may be a function of ligand leaching. [17]

The high product pool volumes expressed by the Rapid A device and consequent dilute mAb concentration may present volume handling complications where large batch sizes are involved. The increased elution volume is likely a combination of mAb desorption kinetics on the device, dead volume in the capsule, and system hold-up volume. Such challenges could be addressed on the vendor side via ligand selection and the design of units with smaller hold-up volumes, or the application of single-pass tangential flow filtration systems by process scientists to reduce product bulk volumes.

The purification of 100 L of CH on a 75 mL Sartobind^®^ Rapid A device using the MU-RCC system is, to our knowledge, the first of its kind for mAb purification at pilot scale. The pilot-scale device was exposed to a total of 57 purification cycles with an aggregate duration of circa 19 h. By comparison, a 900 mL PrismA column would be required to process the same amount of material over four purification cycles for a duration of approximately 14 h, not accounting for column qualification and pre- and post-sanitization activities. The adsorbent volume requirements coupled with capacity utilisation would present cost advantages in the case of the membrane format. The 45 g/L load density used for bench-scale studies appeared to be at the limits of the dynamic binding capacity of the membrane device, which was more apparent for the first lot of pilot-scale runs. On adjustment of the load density to 35 g/L, the process and product attributes were comparable between the 1 mL and 75 mL membrane devices, demonstrating scalability.

We considered a process-scale scenario for the capture of a 2000 L mAb batch with similar titres to CH1–CH5. Table 6 shows parameter estimates for selected chromatography media volumes for the Sartobind^®^ Rapid A membrane and the high-capacity MabSelect™ PrismA resin. Values for the PrismA resin are based on our validated 2000 L scale manufacture of this mAb. Rapid A MVs of 0.8 L, 1.6 L, and 3.2 L were considered and compared to a 32 L MabSelect™ PrismA column. It is noteworthy that the scale-up of a Rapid A device can be achieved using either large-volume capsule formats or stacks of modular cassette formats [17]. The load densities were adjusted to accommodate processing of the entire batch, which is reflected in the corresponding cycle numbers.

When using the membrane (40× less ProtA media, 17% less product load per litre, and 10× shorter cycle time), a significant number of purification cycles would be required to process 4 kg of mAb, totalling approximately 48.6 h in duration for a 0.8 L Rapid A. The high cycle count, albeit with lifetime expectation, results in up to 73× improvement in ProtA media capacity utilisation per batch with an expected 10× gain in productivity compared to the resin. The high productivity in the membrane scenario comes at the expense of buffer usage and step duration which are 2.5× and 4× that of the resin, respectively.

With the improved product purity levels generated by the membrane, the further refinement of downstream polishing and clarification steps could be made redundant. Further studies are required, as this would reduce the overall buffer consumption impact between membrane- and resin-based purification schemes. The small facility footprint and single-use application of this technology may be suited to a multi-product facility.

## 5. Conclusions

The Sartobind^®^ Rapid A membrane is a scalable alternative to conventional ProtA resins for the isolation and purification of mAbs from CH. Productivity gains (10×) and improved capacity utilisation of ProtA media in the membrane format may partially address constraints for high-titre feeds if larger membranes combined with fit-for-purpose chromatographic equipment can be developed. This single use of a smaller MV may present cost benefits where only a few production batches are required per year to serve a niche market, e.g., orphan-status rare diseases. The purity of product pools expressed by the Rapid A device may be advantageous to reduce downstream polishing steps, which could boost process yields and reduce downstream production times. The single-use proposition of the Rapid A membrane also mitigates concerns around column handling, including the integrity of the packed bed and the risk of bioburden and batch-to-batch contamination, which in turn could improve production times.

## Figures and Tables

**Table 1:** mAb batches used for the capture chromatography experiments.

Batch No.	mAb Titre (g/L)	Batch Size (L)	Total mAb (g)
CH1	1.7	500	850
CH2	1.9	5	9.5
CH3	1.7	500	850
CH4	1.8	200	360
CH5	1.8	50	90

**Table 2:** Buffers used for the chromatography experiments. Buffer composition is proprietary to CSL Innovation Pty Ltd.

Buffer	Application	pH
PBS	Equilibration, post-load wash	7.4 ± 0.1
Wash 1	High pH wash	8.5 ± 0.1
Wash 2	Intermediate pH wash	5.5 ± 0.1
Elution-buffer	Elution at low pH	3.6 ± 0.1
Regen-buffer	Regeneration/clean-in-place	13.0 ± 0.5

**Table 3:** Volume and flowrate consideration during chromatography cycle for mAb capture with Sartobind^®^ Rapid A membrane and MabSelect™ PrismA resin.

Device	Sartobind^®^ Rapid A		MabSelect™ PrismA Resin	
Phase	Volume (MV)	Flow Rate (MV/min)	Volume (CV)	Flow Rate (CV/min)
Equilibration	8	10	3	0.25
Protein load (g/L)	45 ^a^	5 ^b^	50 ^a^	0.25
Post-load wash	8 ^c^	10	5	0.25
Wash 1	8	10	5	0.25
Wash 2	8 ^d^	10	5	0.25
Elution	15	5	5	0.25
Post-elution wash	4	10	n/a	n/a
Regeneration	10 ^e^	3	3	0.25
Re-equilibration	5 ^f^, 5	3 ^f^, 10	3.0	0.25

^a^ For Sartobind^®^ Rapid A DBC determination experiments, load density was 90 g/L. For cycling experiments, load density of Sartobind^®^ Rapid A (1 mL) was 45 g/L. For MabSelect™ PrismA resin DBC determination experiments, load density was 73 g/L. For other resin experiments, load density was 50 g/L. ^b^ For DBC experiments, product load flow rate was varied as described in preceding sections. For regular protein runs, 5 MV/min was chosen as the optimal flow rate as it delivered improved product quality whilst maintaining a fast flow rate. ^c^ In scale-up runs, post-load wash was extended by 500 mL to displace 75 mL membrane housing hold-up volume and 200 mL MU-RCC system hold-up volume. ^d^ In scale-up runs on MU-RCC unit, Wash 2 was extended to reach UV absorbance less than 20 mAU. ^e^ Upflow was applied for regeneration phase. Other phases prior to regeneration were performed in downflow direction. ^f^ Upflow was applied for the first 5 MV re-equilibration phase at 3 MV/min. Then, further 5 MV in downflow direction at 10 MV/min.

**Table 4:** Comparison of typical DBC, yield, cycle time, productivity, and mean and standard deviation values (*n* = 3) of product quality attributes between Rapid A membrane (1 mL) and MabSelect™ PrismA resin (4.7 mL), using CH2 as the source load material.

Parameter	Sartobind^®^ Rapid A	MabSelect™ PrismA
DBC_10%_ (g/L)	58.8	80.4
Load Density (g/L)	45	50
Residence Time (min)	0.2 ^a^	4.0
Cycle Time (h)	0.28	3.78
Eluate Concentration (g/L)	3.8 ± 0.0	26.7 ± 0.4
Eluate Volume (MV|CV)	11.0 ± 0.0	1.9 ± 0.0
mAb Recovery (%)	91.1 ± 0.4	98.1 ± 0.3
Monomer (%)	99.1 ± 0.0	95.4 ± 0.6
Aggregate Reduction (%)	90.7 ± 0.0	41.5 ± 9.1
HCP Clearance (LRV)	2.8 ± 0.0	2.2 ± 0.1
HCP species detected	1	22
hcDNA Clearance (LRV)	4.7	2.5
Residual ProtA (ppm)	1.4	5.0
Productivity (g/L/h)	146.4 ± 0.4	13.0 ± 0.3

^a^ Used during product load and elution phases. See Table 3 for sequence.

**Table 5:** Comparison of 1 mL and 75 mL Sartobind^®^ Rapid A membrane devices with respect to eluate quality and performance attributes using the same starting feedstock (CH5). Additionally, 1 cycle was run on the 1 mL device against 29 on the 75 mL device at a load density of 35 g/L. The data shown is that of the pooled product bulk from the purification run(s) for each device.

Attributes	Sartobind^®^ Rapid A Membrane	
	1 mL	75 mL
Load Density (g/L)	35.0	35.0
mAb Recovery (%)	91.4	89.8 ± 0.5
Monomer (%)	98.8	98.6
Aggregate Reduction (%)	91.7	90.3
HCP Clearance (LRV)	2.8	2.6
hcDNA Clearance (LRV)	4.7	4.3
Eluate Concentration (g/L)	4.0	3.7 ± 0.2
Eluate Volume (MV)	8.4	8.9 ± 0.5

**Table 6:** Parameter estimates for larger membrane sizes vs. resin column to process one 2000 L batch of CH, with mAb titre of 2 g/L, at manufacturing scale.

Parameter	Sartobind^®^ Rapid A			MabSelect™ PrismA
	0.8 L	1.6 L	3.2 L	32.0 L
Load density (g/L)	35	35	35	42
Cycle time (h)	0.34	0.34	0.34	3.89
Purification cycles	143	72	36	3
Capacity utilisation per batch (%) ^a^	95	48	30	1.3
Max. flow rate (L/h)	480	960	1920	600
Processing time (h)	48.6	24.5	12.2	11.7
Expected Yield (%)	90	90	90	98
Buffer consumption (L)	8122 ^b^	8179 ^b^	8179 ^b^	3200 ^c^
Productivity (g/L/h)	102.9	91.8	92.2	10.5

^a^ Assuming a membrane|resin lifetime capacity of 150 cycles. ^b^ Extended wash to replace membrane dead volume (approximately 2.5 times MV) is included. Pump washes were included. ^c^ One cycle each of pre- and post-use sanitization, and column qualification was included for resin-based process.

## Data Availability

All relevant data are contained in this article.
